# Multidisciplinary Evaluation of Interstitial Lung Diseases: New Opportunities Linked to Rheumatologist Involvement

**DOI:** 10.3390/diagnostics10090664

**Published:** 2020-09-02

**Authors:** Enrico De Lorenzis, Silvia Laura Bosello, Francesco Varone, Giacomo Sgalla, Lucio Calandriello, Gerlando Natalello, Bruno Iovene, Giuseppe Cicchetti, Laura Gigante, Lucrezia Verardi, Elisa Gremese, Luca Richeldi, Anna Rita Larici

**Affiliations:** 1 Institute of Rheumatology, Catholic University of the Sacred Heart, 00168 Rome, Italy; delorenzis.e@gmail.com (E.D.L.); gerlando.natalello@gmail.com (G.N.); laura.gigante.91@gmail.com (L.G.); lucreziaverardi@gmail.com (L.V.); elisa.gremese@unicatt.it (E.G.); 2 Ph.D. program in Biomolecular Medicine-Cycle XXXV, University of Verona, 37129 Verona, Italy; 3 Divisione di Reumatologia, Fondazione Policlinico Universitario A. Gemelli IRCCS, 00168 Rome, Italy; 4 UOC Pneumologia, Fondazione Policlinico Universitario A. Gemelli IRCCS, 00168 Rome, Italy; francesco.varone@policlinicogemelli.it (F.V.); giacomo.sgalla@policlinicogemelli.it (G.S.); bruno.iovene@policlinicogemelli.it (B.I.); luca.richeldi@unicatt.it (L.R.); 5 Department of Diagnostic Imaging, Oncological Radiotherapy, and Hematology, Fondazione Policlinico Universitario A. Gemelli IRCCS, 00168 Rome, Italy; lucio.calandriello@policlinicogemelli.it (L.C.); cicchetti.giuseppe88@gmail.com (G.C.); annarita.larici@unicatt.it (A.R.L.); 6 Department of Radiological and Hematological Sciences, Section of Radiology, Catholic University of the Sacred Heart, 00168 Rome, Italy

**Keywords:** interstitial lung disease, systemic rheumatic autoimmune disease, connective tissue disease, rheumatoid arthritis, interstitial pneumonia with autoimmune features, multidisciplinary team

## Abstract

Multidisciplinary team (MDT) discussion is the gold standard in the management of interstitial lung disease (ILD). The rheumatologist is not routinely involved in MDT, even if up to 20% of ILD are related to systemic autoimmune rheumatic diseases (SARD). The study aims to assess the agreement and its variation over time between rheumatologists and pulmonologists in the screening of SARD and between rheumatologists and an MDT extended to rheumatologists (eMDT) in evaluating the progression of SARD. We computed the agreement between the pulmonologist and rheumatologist in the identification of red flags for SARDs of 81 ILD cases and between the rheumatologist alone and eMDT in the confirmation of 70 suspected SARD-ILD progressions. The agreement between rheumatologists and pulmonologists was moderate for the detection of autoimmunity test positivity (*κ* = 0.475, *p* < 0.001) and family history of SARD (*κ* = 0.491, *p* < 0.001) and fair for the identification of extrapulmonary symptoms (*κ* = 0.225, *p* = 0.064) or routine laboratory abnormalities consistent with SARD. The average agreement between the rheumatologist and eMDT in the identification of ILD progression was moderate (*κ* = 0.436, *p* < 0.001). The class of agreement improved from the first to the third semester. The average agreement with the rheumatologist ranged from fair to moderate, suggesting that a shared evaluation of SARD-ILD in eMDT could improve the diagnostic work-up and the evaluation of ILD progression.

## 1. Introduction

Diffuse interstitial lung disease (ILD) is a heterogeneous group of pulmonary diseases with high morbidity and mortality rates [1]. Up to 20% of ILD may be related to systemic autoimmune rheumatic diseases (SARD), primarily connective tissue diseases, rheumatoid arthritis and systemic vasculitis [2]. Making an accurate diagnosis of a specific cause of ILD is often challenging but it is undoubtedly crucial, since the treatment may radically change [3]. Multidisciplinary team (MDT) evaluation has been proposed as the gold standard [4] in the management of ILD. Nonetheless, there are no formal recommendations for its composition and working process [5]. Even if current guidelines consider it mandatory to exclude a systemic autoimmune rheumatic disease (SARD) in any newly diagnosed ILD, a rheumatologist is not routinely involved in MDT. Usually, a rheumatology consultation is advised only when the referring physician recognizes symptoms, laboratory tests or any other clinical clue consistent with SARD. This approach has shown substantial limitations [6] because of the inter-individual expertise of the physician in the identification of extrapulmonary manifestation of SARD and a partial agreement about which serological tests to perform in order to exclude a SARD. On the other side, when a SARD-ILD is diagnosed, given its systemic involvement, most of the decisions about its management are entrusted to the rheumatologist. In patients with SARD, the symptoms of lung involvement are often non-specific and the correct identification of progression of ILD on HRCT (high-resolution computed tomography) mainly drives the therapeutic choices.

This observational study analyzed consecutive cases that are discussed in an MDT that included rheumatologists for a suspected or definite ILD secondary to SARDs, in a large Italian tertiary care university hospital. We assessed the level of agreement between the pulmonologist and rheumatologist in the identification of red flags of SARD in the screening phase and between the rheumatologist and eMDT in the assessment of progression during follow-up.

## 2. Methods

### 2.1. Multidisciplinary Team Composition and Activity

MDT meetings were usually held weekly to discuss newly diagnosed ILD or to re-evaluate already known ILD patients during follow-up at our institution. The minimum attendance of the conventional MDT (cMDT) included two pulmonologists, two chest radiologists with specific expertise in ILD and a pathologist if a lung biopsy was available. Two rheumatologists joined the discussion twice a month in an extended multidisciplinary team (eMDT) meeting. The patients discussed in eMDT had ILD and a suspected or established diagnosis of SARD. Prior to any eMDT, a pulmonologist and a rheumatologist already independently evaluated every case. We retrospectively evaluated the data of clinical cases discussed in an eMDT over a period of 18 months—from 1 January 2018 to 30 June 2019. The final eMDT decisions on ILD diagnosis or progression and any modification of treatment were recorded. The study protocol was approved by the Ethics Committee of Fondazione Policlinico Universitario A. Gemelli (protocol no. 0027493/20—7 January 2020).

### 2.2. Selection of Cases to Be Discussed at eMDT

**Figure 1:**
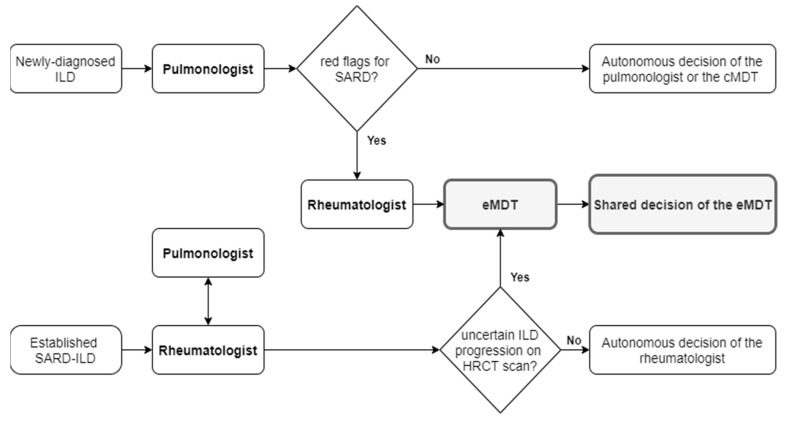
Flowchart of the selection of cases to be discussed at eMDT. ILD: interstitial lung disease; SARD: systemic autoimmune rheumatic disease; cMDT: conventional multidisciplinary team; eMDT: multidisciplinary team extended to rheumatologists; SARD-ILD: interstitial lung disease related to systemic autoimmune rheumatic disease; HRCT: high-resolution computed tomography.

Two groups of ILD cases were discussed at eMDT meetings, defined as group D (eMDT discussion aimed at diagnosing or ruling out a SARD-ILD) and group P (eMDT discussion aimed at defining or excluding ILD progression in a known SARD-ILD). The flowchart that guided the choice to discuss a clinical case at eMDT by pulmonologists and rheumatologists is shown in Figure 1.

Group D included patients who presented to the ILD unit of the Fondazione Policlinico Universitario A. Gemelli and had a suspected diagnosis of ILD related to SARD. The diagnosis of SARD was usually suspected by the pulmonologists because of the detection of one or more red flags. These included possible extrapulmonary symptoms of SARD, family history of SARD, the positivity of autoimmunity tests or abnormalities of routine laboratory tests consistent with extrapulmonary involvement of SARD. All the laboratory tests were carried out according to the standard practice of the center. The patients suspected to have a SARD were referred to a dedicated outpatient rheumatological clinic of the Fondazione Policlinico Universitario A. Gemelli. Complete physical examination was performed, and personal and family history were collected for each patient. Additional laboratory tests were prescribed when needed, according to standard clinical practice, to confirm or rule out the diagnosis of SARD. These clinical cases were finally discussed at eMDT to define the most probable etiology of ILD and eventually confirm a SARD-ILD and decide the most appropriate management.

Patients of group P were referred to eMDT discussion by the rheumatologist. Group P included patients regularly followed at the outpatient rheumatology clinic, with established SARD-ILD and an uncertain progression of the pulmonary disease based on symptoms, functional data and HRCT scan. The discussion aimed to define the presence of progression of SARD-ILD according to both available clinical information, functional lung parameters and comparison of two or more longitudinal HRCT exams. Patients with superimposed conditions that could not be directly associated with the SARD—including infections, neoplasms, environmental diseases or HRCT artefacts limiting a correct interpretation of images—were not considered to be progressors. Disease duration from ILD diagnosis, pulmonary function tests (PFTs), occupational exposures and full medical and drug history were available for all patients. The Charlson comorbidity index (CCI) [7] was calculated as a summary of comorbidity burden [8].

### 2.3. Statistical Analysis

Data were analyzed using SPSS Statistics version 26.0 for Windows (IBM Corp., Armonk, NY, USA). Continuous variables were reported as mean ± SD or median with interquartile range (IQR), according to the distribution of the data, while categorical variables were reported as numbers and percentages. Analysis of categorical variables was performed with the *χ^2^* test or Fisher’s exact when appropriate, and comparisons between groups of continuous variables were performed with the Mann–Whitney *U* test or *t*-test, according to the data distribution. Statistical significance was defined as *p* < 0.05. The level of agreement between the pulmonologist and rheumatologist on the presence of single red flags and progression was evaluated using Cohen’s kappa coefficient (*κ*) and results were reported with a 95% confidence interval. The degree of agreement was termed according to semiquantitative Landis and Koch interpretation of Cohen’s *κ* [9]. Positive and negative predictive values of the pulmonologist and rheumatologist in the identification of red flags for SARD and progression of ILD, respectively, were computed. Subgroup analysis of inter-rater agreement based on activity of eMDT meetings over three consecutive six-month periods was performed to determine whether the level of agreement changed over time.

## 3. Results

### 3.1. Characteristics of the Patients

The clinical characteristics of the patients enrolled are shown in Table 1. One-hundred fifty-one cases were discussed at the eMDT over a period of 18 months. Eighty-one (53.6%) patients were in group D and 70 (46.4%) in group P, respectively. Patients of group D had a shorter disease duration (median 0.6 vs. 2.0 years, *p* = 0.036) and needed more frequent oxygen supplementation at home (28.6% vs. 12.3%, *p* = 0.049), while they were comparable to group D with regard to mean age, male to female ratio, smoke exposure, CCI score, FVC, DL_CO_ and proportion of nonspecific interstitial pneumonia (NSIP), usual interstitial pneumonia (UIP) and organizing pneumonia (OP) pattern on HRCT scan.

### 3.2. Evaluation of Patients with Suspected SARD-ILD (Group D)

All the patients of group D were required to have at least one red flag for SARD in order to be referred to a rheumatologist and be included in the study. Overall, 45 patients (55.6%) had one red flag, 30 (37.0%) had two red flags and 6 (7.4%) had three red flags according to the evaluation of the pulmonologists. At the time of first evaluation, sixty-eight patients (84.0%) complained of respiratory symptoms; in particular, 59 (72.8%) had dyspnea, 43 (53.1%) had cough, 8 (9.9%) had nail clubbing and 3 (3.7%) had hemoptysis.

All the patients had their first rheumatological evaluation within two weeks of the pulmonologists’ evaluation. During rheumatological evaluation, medical history and physical examination revealed symptoms consistent with SARD and not explained by a known concomitant condition in 64 patients (79.0%). In particular, 39 patients (48.1%) complained of arthralgias which were most severe in the early morning or associated with swelling or prolonged morning stiffness, 23 (28.4%) had Raynaud’s phenomenon, 22 (27.2%) xerophthalmia, 18 (22.2%) xerostomia, 17 (21.0%) photosensitivity or other inflammatory skin lesions, 16 (19.8%) distal paresthesia, 12 (14.8%) puffy hands, 8 (9.9%) dysphagia, 7 (8.6%) myalgias, 4 (4.9%) recurrent fever, 4 (4.9%) history of thrombosis, 3 (3.7%) history of miscarriage, 3 (3.7%) acral ulcers and 2 (2.5%) recurrent oral aphthosis. Family history of SARD was reported by 10 (12.3%) patients. The rheumatologists implemented the screening information provided by the pulmonologist with additional tests in all the patients of group D. In particular, 36 patients (44.4%) had additional routine blood or urine tests, 53 (65.4%) completed or repeated the available autoimmunity tests, 18 (22.2%) had an articular X-ray or ultrasound, 9 (11.1%) an electromyography, 35 (43.2%) a nailfold capillaroscopy, 16 (19.8%) a biopsy of the minor salivary glands of the lip and 11 (13.6%) an ophthalmologic evaluation for lacrimal function. A lung biopsy was deemed necessary to obtain the final diagnosis only in one case with an indeterminate pattern on HRCT after a preliminary multidisciplinary evaluation.

The rheumatologist with the implementation of all tests revealed that abnormalities of routine tests consistent with SARD were found in 44 (54.3%) patients overall. Specifically, 40 patients (49.4%) had persistent high C reactive protein or erythrocyte sedimentation rate, 13 (16.0%) had high creatine kinase or aldolase and 10 (12.3%) had high creatinine or abnormal urine sediment. Finally, 62 patients (76.5%) had abnormal autoimmunity tests, specifically antinuclear antibodies (ANA) were positive in 50 (61.7%) patients, anti-extractable nuclear antigen (ENA) in 21 (25.9%), rheumatoid factor in 17 (21.0), anti-neutrophil cytoplasmic antibodies (ANCA) in 9 (11.1%), anti-citrullinated protein antibodies (ACPA) in 6 (7.4%) and anti-double stranded DNA (dsDNA) in 2 (2.5%). Low levels of complement fractions (C3 or C4) were found in four (4.9%) patients. After the rheumatological referral, 22 (27.2%) more patients were reported to have symptoms consistent with SARD, but the presence of such suspicious clinical manifestation was not confirmed for six patients (7.4%). Previously unknown positivity of autoimmunity tests and abnormalities of routine tests consistent with SARD were reported after rheumatological evaluation in 10 (12.3%) and 38 (46.9%) more patients, respectively. Abnormalities of laboratory analysis were not confirmed after retesting in seven patients (8.6%) and one patient for routine and autoimmunity tests, respectively. Lastly, six (7.4%) more patients reported family history of SARD during rheumatological evaluation and one patient did not confirm their grandmother’s history of rheumatoid arthritis. In group D, there was moderate agreement between rheumatologists and pulmonologists for the identification of immunity test positivity (*κ* = 0.475, CI 95% 0.248–0.723, *p* < 0.001) and family history of SARD (*κ* = 0.491, CI 95% 0.173–0.806, *p* < 0.001). On the contrary, there was fair agreement for the identification of extrapulmonary symptoms (*κ* = 0.225, CI 95% 0.025–0.425, *p* = 0.064) and poor agreement for the identification of routine laboratory abnormalities consistent with SARD (*κ* = 0.101, CI 95% −0.007–0.209, *p* = 0.081) (Table 2). Positive and negative predictive values of the pulmonologist in the identification of each group of red flags compared to rheumatological evaluations are reported in Table 2.

**Figure 2:**
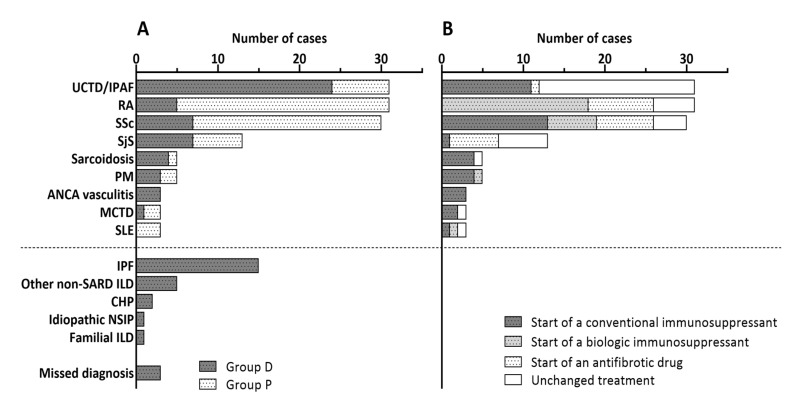
ILD cause of group D and group P (**A**) and therapeutic program of SARD-ILD (**B**) after discussion at eMDT. UCTD: undifferentiated connective tissue disease; IPAF: idiopathic pneumonia with autoimmune features; RA: rheumatoid arthritis; SSc: systemic sclerosis; PM: polymyositis; ANCA: antineutrophil cytoplasmic antibodies; MCTD: mixed connective tissue disease; SLR: systemic erythematosus lupus; SARD: systemic autoimmune rheumatic disease; ILD: interstitial lung disease; CHP: chronic hypersensitivity pneumonitis; NSIP: nonspecific interstitial pneumonia; eMDT: multidisciplinary team extended to rheumatologists.

After rheumatological evaluation, the eMDT diagnosed in 54 patients (66.7%) a SARD-ILD. In particular, an undifferentiated connective tissue disease or interstitial pneumonia with autoimmune features [10] was diagnosed in 24 patients (29.6%), systemic sclerosis in 7 (8.6%), Sjögren syndrome in 7 (8.6%), rheumatoid arthritis in 5 (6.2%), sarcoidosis with extrapulmonary involvement in 4 (4.9%), ANCA vasculitis in 3 (3.7%) and mixed connective tissue disease in 1 patient (1.2%). Of note, since the borders between undifferentiated connective tissue disease with ILD and interstitial pneumonia with autoimmune features are still not completely defined, these diagnoses were considered together. Twenty-two patients (27.2%) received a diagnosis of primary pulmonary disease; more specifically, 15 (18.5%) of idiopathic pulmonary fibrosis, 2 (2.5%) of chronic hypersensitivity pneumonia, 1 (1.3%) of familial ILD, 1 (1.3%) of idiopathic NSIP and 5 (11.1%) of other pulmonary diseases non-related to SARD. Finally, eMDT discussion was inconclusive for three patients (3.7%) who were committed to close follow-up and repetition of specific tests (Figure 2A).

### 3.3. Evaluation of Patients with an Uncertain Progression of SARD-ILD (Group P)

In group P, 26 patients (37.1%) had rheumatoid arthritis, 23 (32.9%) systemic sclerosis, 7 (10.0%) undifferentiated connective tissue disease or interstitial pneumonia with autoimmune features, 6 (8.6%) Sjögren syndrome, 3 (4.3%) systemic lupus erythematosus, 2 (2.8%) polymyositis and 1 (1.4%) sarcoidosis with extrapulmonary involvement (Figure 2A). Among the 70 patients, the progression of ILD related to SARD was considered to be certain or probable for 37 patients (52.9%) and was excluded or considered to be unlikely for 33 patients (47.1%) by the rheumatologist before eMDT discussion. After eMDT discussion, based on clinical and functional data and HRCT examination, 27 (38.6%) patients were recognized as progressors. Overall, 5 (7.1%) progressors and 15 (21.4%) non-progressors according to eMDT were misdiagnosed by the rheumatologist alone. The agreement between rheumatologist and eMDT for the identification of ILD progression was moderate (*κ* = 0.436, CI 95% 0.234–0.638, *p* < 0.001). Positive and negative predictive values of rheumatological identification of lung disease progression compared to eMDT conclusion were 59.5% and 84.8%, respectively.

### 3.4. Modification in the Agreement in Group D and P over Time

**Figure 3:**
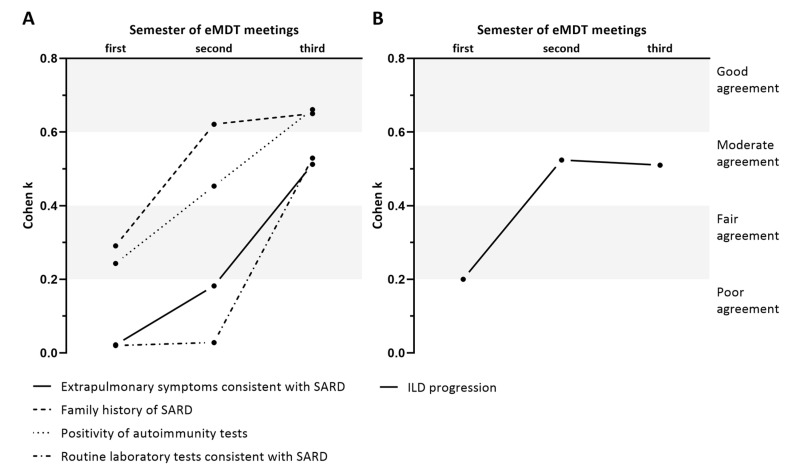
Level of agreement over three consecutive six-month periods between pulmonologist and rheumatologist in the identification of red flags of SARD (**A**) and between rheumatologist and eMDT in the definition of ILD progression (**B**). eMDT: multidisciplinary team extended to rheumatologists; SARD: systemic autoimmune rheumatic disease; ILD: interstitial lung disease.

The level of the agreement between rheumatologist and pulmonologist changed over time. As shown in Figure 3A, the degree of agreement improved from the first to the last six-month period for the detection of the red flags. In particular, the level of agreement improved from fair to good in the detection of positive autoimmunity tests and family history of SARD and from poor to moderate in the detection of symptoms or routine laboratory tests consistent with SARD. Similarly, Figure 3B shows that the agreement between rheumatologist alone and eMDT increased from poor to moderate in the same frame of time as the identification of ILD progression.

### 3.5. Comparison of Patients with SARD-ILD Observed or Treated (Therapy Initiation or Change)

The total number of patients with a previous or new diagnosis of SARD-ILD was 124. In particular, 31 (25.0%) had rheumatoid arthritis, 31 (25.0%) undifferentiated connective tissue disease or interstitial pneumonia with autoimmune features, 30 (24.2%) systemic sclerosis, 13 (10.5%) Sjögren syndrome, 5 (4.9%) polymyositis, 5 (4.0%) sarcoidosis with extrapulmonary involvement, 3 (2.4%) mixed connective tissue disease, 3 (2.5%) ANCA vasculitis and 3 (2.5%) systemic lupus erythematosus. Therapeutic strategy changed after eMDT in 72 patients (55.5%). Specifically, a standard immunosuppressive drug (azathioprine, cyclophosphamide or mycophenolate mofetil with or without at least 0.5 mg/kg of daily prednisone) was prescribed in 50 (40.3%) patients, a biotechnological immunosuppressant (rituximab, abatacept or tocilizumab) in 13 (10.3%) and an antifibrotic drug (nintedanib or pirfenidone) in 9 (7.3%). Patients who started or changed treatment had lower FVC (81.0 ± 21.4% vs. 90.0 ± 27.1%, *p* = 0.05) compared to patients who continued the observation without treatment modifications. There were no other significant differences among groups that modified the treatment or did not. Being part of group D or P, male to female ratio, mean age, smoking habit, comorbidity burden according to CCI, duration of ILD, DL_CO_, prevalence of NSIP or UIP pattern and oxygen therapy were all comparable between the two groups (Table 3). The therapeutic choices of eMDT according to specific diagnosis of SARD-ILD are plotted in Figure 2B. Therapeutic strategy was different according to the diagnosis and, in particular, patients with undifferentiated connective tissue disease (UCTD)/idiopathic pneumonia with autoimmune features (IPAF) were less likely to have their treatment modified compared to those with a more specific diagnosis.

## 4. Discussion

Multidisciplinary discussion has been implemented in clinical practice for a wide range of diseases. In particular, MDT plays a crucial role in the management of diseases or procedures with high mortality rates given that MDT meetings imply an adequate investment in human resources. The official ATS/ERS/JRS/ALAT clinical practice guidelines recommend a dynamic process of MDT in the management of ILD since individual decisions on complex cases may increase the risk of delayed diagnosis, inappropriate treatment or unnecessary additional diagnostic testing [4]. Although one ILD case out of five can be related to SARD, a recent systematic evaluation of diagnostic ILD practice around the world showed that only 37.1% of MDT cases include a rheumatologist [5]. The clinical impact of this practice is still largely unknown.

To our knowledge, this paper is the first description of the real-life decision-making flow of an MDT that included a rheumatologist. Broadly speaking, our data showed that the average agreement between pulmonologist and rheumatologist in the identification of red flags for SARDs ranges from fair to moderate. It can be therefore deduced that the involvement of the rheumatologist in the multidisciplinary discussion about patients with suspected SARD provides more comprehensive clinical information that could lead to a more accurate diagnosis. Similarly, the average agreement between rheumatologist and eMDT in the identification of ILD progression is moderate. This observation indicates that a collegial discussion of challenging cases of SARD-ILD could improve the assessment of lung disease compared to the single rheumatologist’s evaluation, with potential impact on therapeutic choices.

The information related to group D focuses on the diagnostic phase of the ILD. In our cohort of patients with newly diagnosed ILD that have been referred to a rheumatologist, some clinically important details were potentially overlooked during the individual evaluation by the pulmonologist. In particular, the relationship between positive and negative predictive values indicates that the pulmonologist physician is more accurate in the identification of a red flag than in the exclusion of its presence. The current guidelines for idiopathic pulmonary fibrosis (IPF) diagnosis do not recommend a rheumatological referral for every patient with newly diagnosed ILD, but only for those with suggestive clinical manifestations, serologies or other characteristics atypical for idiopathic interstitial pneumonia, without further defining the specific characteristics that need to be checked. Moreover, serological testing is recommended in all patients with newly identified ILD, but specific routine or autoimmunity tests which have to be investigated are not fully addressed and largely performed on a case-by-case basis according to associated symptoms and signs [4]. Since clinical manifestation of SARD could be underestimated by a non-rheumatologist physician, the decision of the rheumatological referral is therefore totally entrusted to the experience of the single pulmonologist. It is also important to highlight that the information provided by positivity or negativity of a single screening test is often overestimated. In fact, autoantibodies could be present in patients with non-rheumatic diseases and even in healthy subjects. It is noteworthy that both idiopathic pulmonary fibrosis and positivity of autoantibodies in healthy patients are more frequent in late adulthood. Moreover, variability in the results may be a consequence of discrepancies among laboratories and techniques [11]. Therefore, clinicians should be cautious in the interpretation of both positive and negative results. A comprehensive evaluation of all the available data is therefore the only thorough way to make an accurate diagnosis.

The analysis of group P reveals the limitations of individual rheumatologic management of SARD-ILD. The information provided by HRCT scans is often crucial in the decision-making process since pulmonary manifestation can be mimicked by extra-pulmonary manifestations (and vice versa) in patients with SARD. Characterization and quantification over time of ILD on HRCT is therefore a crucial issue in both diagnosis and management of the disease. Nevertheless, due to the heterogeneity and complexity of ILD patterns, these processes can be challenging, with a variable degree of inter- and intra-observer reproducibility [12,13,14] even among expert radiologists [15]. In the cohort of the patients whose ILD progression was considered uncertain by the rheumatologist based on clinical and HRCT information, at least one out of five patients is potentially misdiagnosed. The relationship between positive and negative predictive values indicates that the rheumatologist is more accurate in the exclusion of ILD progression than in its identification.

The subgroup analysis shown in Figure 3 demonstrates that the level of the agreement between specialists increased over time. In particular, the level of agreement between pulmonologist and rheumatologist in the identification of red flags for SARD and between rheumatologist and eMDT in the identification of ILD progression improved from the first to the last six-month period. Interestingly, similar results have been also observed in the longitudinal diagnostic assessment of idiopathic interstitial pneumonias [16].

This suggests that eMDT feedback can improve the diagnostic performance in the clinical evaluation [17] and interpretation of HRCT scans [18] in ILD, as previously suggested. As explained below, the potential advantages for diagnostic accuracy or clinical outcomes of this improvement can be hypothesized but not be directly inferred from this study. It is important to highlight that, since eMDT is feasible only for selected SARD-ILD cases, an appropriate referral based on a preliminary evaluation of the physician is crucial, but a comprehensive MDT discussion provides information that goes far beyond the simple definition of etiology and progression of ILD.

Lastly, age and comorbidity are sometimes restrictions to the prescription of both immunosuppressants and anti-fibrotic drugs. We observed that patients committed to observation had higher FVC compared to those committed to a modification of the treatment but—surprisingly—the two groups did not differ according to age or comorbidity burden. It can be therefore inferred that eMDT discussion with robust conclusions could promote a personalized treatment even in more complex patients.

This study has some limitations. Firstly, it is a single-group/single-center experience and practice of other MDTs could be markedly different worldwide. Secondly, the selection of the patients included in the analysis could have influenced the results. The data address a cohort of patients that are brought in discussion at eMDT by pulmonologists or rheumatologists. The prevalence of missed diagnosis of SARD-ILD or progression of ILDs in the patients not discussed in eMDT remains unknown since control groups were not available. However, because eMDT is commonly reserved for more complex cases, this proportion could be acceptably small or less clinically significant. Moreover, laboratory screening tests were not homogeneous in all patients and were supplemented by the rheumatologist for most patients, according to single clinical features. Therefore, any significant inference about the power of laboratory or clinical tools to predict a diagnosis of SARD in eMDT cannot be made. Finally, although our data suggest that eMDT can improve patient care, the impact on survival is not addressed. Longitudinal studies are needed to clarify this aspect and define shared criteria for rheumatologic referral to ensure higher appropriateness. A proper MDT discussion of SARD-ILD patients should be promoted, particularly before the prescription of potentially dangerous and high-cost pharmacological therapies. To date, translational studies exploring progressive ILD have provided new data that show that IPF, SARD-ILD and IPAF represent a continuum of lung damage with shared pathophysiological mechanisms which are, at least in part, independent of nosological classification [19]. Waiting for reliable biomarkers, the multidisciplinary interpretation of clinical, radiographic and laboratory data is currently the most appropriate approach to recognize subtypes of ILD that could benefit from a specific available treatment.

## 5. Conclusions

The average agreement between the rheumatologist and the other specialists in the identification of red flags of SARD and the assessment of progression ranged from fair to moderate and increased in the follow-up. An eMDT discussion can indeed provide a wider availability of clinical information to make clinical decisions in both diagnostic and follow-up phases. The collegial discussion of SARD-ILD cases in the eMDT could therefore improve the diagnostic work-up and the evaluation of lung disease progression compared to the single rheumatologist’s or cMDT evaluations. Potential advantages of eMDT for the clinical outcomes need to be ascertained with longitudinal case-controlled studies.

## Figures and Tables

**Table 1:** Characteristics of patients discussed at eMDT.

	All Patients	Group D	Group P	*p* *
*N*	151	81	70	-
Age (years), mean ± SD	60.3 ± 14.1	62.1 ± 13.0	59.6 ± 13.7	0.198
Female, *n* (%)	105 (69.5)	56 (69.1)	49 (70.0)	0.908
Current or former smokers, *n* (%)	65 (43.0)	38 (46.9)	26 (37.1)	0.281
CCI, mean ± SD	2.9 ± 1.6	2.9 ± 1.6	3.1 ± 1.6	0.785
ILD duration, years, median (IQR)	1.0 (0.3–4.3)	0.6 (0.3–2.5)	2.0 (0.0–7.9)	0.036
FVC, %, mean ± SD	87.2 ± 25.4	87.6 ± 26.0	86.8 ± 25.0	0.333
FVC ≤ 80%, *n* (%)	84 (55.6)	44 (54.3)	40 (57.1)	0.728
DL_CO_, %, mean ± SD	58.1 ± 21.0	60.5 ± 20.7	55.5 ± 21.3	0.219
DL_CO_ ≤ 50%, *n* (%)	87 (57.6)	45 (56.8)	41 (58.6)	0.825
NSIP pattern on HRCT scan, *n* (%)	63 (41.7)	32 (39.5)	31 (44.3)	0.553
UIP pattern on HRCT scan, *n* (%)	58 (38.4)	28 (34.6)	30 (42.9)	0.296
OP pattern on HRCT scan, *n* (%)	6 (4.0)	4 (4.9)	2 (2.9)	0.514
Indeterminate pattern on HRCT, *n* (%)	24 (15.9)	17 (21.0)	7 (10.0)	0.066
Oxygen therapy, *n* (%)	30 (19.9)	10 (12.3)	20 (28.6)	0.049

eMDT: multidisciplinary team extended to rheumatologists; SD: standard deviation; CCI: Charlson comorbidity index; ILD: interstitial lung disease; IQR: interquartile range; FVC: forced vital capacity; DL_CO_: diffusing capacity for carbon monoxide; NSIP: nonspecific interstitial pneumonia; HRCT: high-resolution computed tomography; UIP: usual interstitial pneumonia; OP: organizing pneumonia. * *p* value was the result of comparisons between patients of group D and group P using Student’s *t*-test or Mann–Whitney *U* test for continuous variables, according to the distribution of variables, and chi square test for categorical variables. *p* < 0.05 was considered significant.

**Table 2:** Level of agreement between pulmonologist and rheumatologist in the identification of red flags for SARD.

Red Flags	Pulmonologist	Rheumatologist	Cohen’s k	Agreement *	CI 95%	*p*	PPV **	NPV ^§^
Positivity of autoimmunity tests, *n* (%)	59 (72.8)	62 (76.5)	0.475	Moderate	0.248–0.723	<0.001	88.1	54.5
Family history of SARD, *n* (%)	5 (6.2)	10 (12.3)	0.491	Moderate	0.173–0.806	<0.001	80.0	92.1
Extrapulmonary symptoms consistent with SARD, *n* (%)	48 (59.3)	64 (79.0)	0.225	Fair	0.025–0.425	0.064	87.5	33.3
Routine laboratory tests consistent with SARD, *n* (%)	7 (8.6)	44 (54.3)	0.101	Poor	−0.007–0.209	0.081	85.7	48.6

SARD: systemic autoimmune rheumatic disease; ILD: interstitial lung disease; CI: confidence interval; PPV: positive predictive value; NPV: negative predictive value. * The degree of agreement was termed according to semiquantitative Landis and Koch interpretation of Cohen’s k; ** Probability that patients with a red flags according to the pulmonologist truly have that red flag after rheumatological evaluation; **^§^** Probability that patients without a red flag according to the pulmonologist truly do not have that red flag after rheumatological evaluation.

**Table 3:** Characteristics of patients with SARD-ILD evaluated at eMDT according to the final decision on treatment.

	All Patients with SARD-ILD	Modification of Treatment	No Modification of Treatment	*p* *
*N*	124	72	52	-
Group D/Group P, *n* (%)	54 (43.5)/70 (56.5)	42(58.3)/30(41.7)	31(59.6)/21(40.4)	0.826
Age (years), mean ± SD	59.6 ± 13.8	59.3 ± 14.5	60.1 ± 12.9	0.732
Female, *n* (%)	87 (70.2)	46 (63.9)	41 (78.8)	0.072
Current or former smokers, *n* (%)	51 (41.1)	30 (41.7)	21 (40.4)	0.826
CCI, mean ± SD	2.9 ± 1.6	2.9 ± 1.6	2.9 ± 1.5	0.940
ILD duration, years, median (IQR)	1.0 (0.1–4.7)	0.5 (0.4–4.6)	0.8 (0.3–2.1)	0.788
FVC, %, mean ± SD	84.9 ± 24.2	81.0 ± 21.4	90.0 ± 27.1	0.050
FVC ≤ 80%, *n* (%)	68 (54.8)	43 (59.7)	25 (48.1)	0.199
DL_CO_, %, mean ± SD	58.8 ± 21.7	58.2 ± 23.8	60.0 ± 17.6	0.692
DL_CO_ ≤ 50%, *n* (%)	72 (58.1)	41 (56.9)	31 (59.6)	0.766
NSIP pattern on HRCT scan, *n* (%)	59 (47.6)	37 (51.4)	22 (42.3)	0.318
UIP pattern on HRCT scan, *n* (%)	45 (36.3)	24 (33.3)	21 (40.4)	0.420
OP pattern on HRCT scan, *n* (%)	5 (4.0)	4 (5.6)	1 (1.9)	0.310
Indeterminate pattern on HRCT, *n* (%)	15 (12.1)	7 (9.7)	8 (15.4)	0.340
Oxygen therapy, *n* (%)	27 (21.8)	18 (25.0)	9 (17.3)	0.332

SARD-ILD: interstitial lung disease related to systemic autoimmune rheumatic disease; eMDT: extended multidisciplinary team; SD: standard deviation; CCI: Charlson comorbidity index; ILD: interstitial lung disease; IQR: interquartile range; FVC: forced vital capacity; DLco: diffusing capacity for carbon monoxide; NSIP: nonspecific interstitial pneumonia; HRCT high-resolution computed tomography; UIP: usual interstitial pneumonia. * *p* value was the result of comparisons between patients intended for treatment or observation after eMDT using Student’s *t*-test or Mann–Whitney *U* test for continuous variables and chi square test for categorical variables. *p* < 0.05 was considered significant.
